# Development and Retranslational Validation of an *In Vitro* Model to Characterize Acute Infections in Large Human Joints

**DOI:** 10.1155/2014/848604

**Published:** 2014-04-30

**Authors:** Ingo H. Pilz, Alexander Mehlhorn, David Dovi-Akue, Elia Raoul Langenmair, Norbert P. Südkamp, Hagen Schmal

**Affiliations:** Department of Orthopedic Surgery, University of Freiburg Medical Center, Hugstetter Straße 55, 79106 Freiburg, Germany

## Abstract

Bacterial infections can destroy cartilage integrity, resulting in osteoarthritis. Goal was to develop an *in vitro* model with *in vivo* validation of acute joint inflammation. Inflammation in cocultivated human synovial fibroblasts (SFB), chondrocytes (CHDR), and mononuclear cells (MNC) was successively relieved for 10 days. Articular effusions from patients with (*n* = 7) and without (*n* = 5) postoperative joint infection in healthy patients (ASA 1-2) were used as model validation. Inflammation *in vitro* resulted in an enormous increase in IL-1 and a successive reduction in SFB numbers. CHDR however, maintained metabolic activity and proteoglycan synthesis. While concentrations of bFGF *in vivo* and *in vitro* rose consistently, the mRNA increase was only moderate. Concurring with our *in vivo* data, cartilage-specific IGF-1 steadily increased, while IGF-1 mRNA in the CHDR and SFB did not correlate with protein levels. Similarly, aggrecan (ACAN) protein concentrations increased *in vivo* and failed to correlate *in vitro* with gene expression in either the CHDR or the SFB, indicating extracellular matrix breakdown. Anabolic cartilage-specific BMP-7 with highly significant intra-articular levels was significantly elevated *in vitro* on day 10 following maximum inflammation. Our *in vitro* model enables us to validate early inflammation of *in vivo* cell- and cytokine-specific regulatory patterns. This trial is registered with MISSinG, DRKS 00003536.

## 1. Introduction

Acute inflammation, a rapid response to infectious microorganisms or injured tissue, culminates in the local recruitment and activation of immune cells. Goal is to resolve the initiating event by making microbes harmless or by removing cellular debris. In case of success, normal tissue architecture is restored by resolving acute inflammation. Otherwise, inflammatory processes will persist and evolve [1].

The most frequent endogenous causes in synovial human joints are hematogenic spread in the wake of a bacteremia [2]. These are mainly triggered by predisposing immunosuppressive factors such as diabetes or alcohol abuse in conjunction with bacteremia [3]. Cartilage, owing to its limited self-healing capacity, is particularly vulnerable [4–7]. After just 6 days of inflammation, irreversible cartilage damage in a rabbit model was observed [8]. The reason for this is the impact on cartilage entailing destruction of the extracellular matrix (ECM) network. Degradation of the ECM, a specialized reservoir of regulatory factors, greatly influences the outcome of inflammatory responses [9, 10]. The consequences are all the more serious as cartilage contains just 1–5% chondrocytes maintaining and regenerating ECM, and their amount declines with age [11–13]. Chondrocytes ensure equilibrium between the synthesis and degradation of their structural components such as collagen type II and proteoglycans. Therefore, the ECM's inflammation and destruction result in an altered chondrocytic phenotype with unrounding, increased proliferation, and altered gene expression [14]. The massive release of proinflammatory IL-1, during acute inflammation, for example, suffices to almost completely suppress collagen type I and II and aggrecan mRNA expression [15, 16]. Altered collagen expression during this process results in “repair” cartilage with inferior biochemical properties [17].

We know very little about early events during acute bacterial inflammation in synovial joints. To elucidate these early events with respect to mediators and biochemical impacts on chondrocytes (CHDR), we established an* in vitro* coculture model comprising crucial cell types in synovial joints. We validated our model using the lavage fluids from acutely postoperatively infected knee joints as reference.

Inflammatory stimuli can be provoked by applying lipopolysaccharides (LPS) derived from gram-negative bacteria [18]. LPS is considered a potent activator of the macrophage secretory response [19]. They are, in addition to neutrophils, key mediators of the host's antimicrobial defence [20]. While LPS application results in a sharp and transient release of IL-1, it is quickly diluted during media exchanges. Phorbol 12-myristate 13-acetate (PMA) application, however, leads to stable, nontransient IL-1 accumulation [21]. Moreover, the PMA challenge shifts the ratio of IL-1 from intracellular towards extracellular accumulation, resulting in elevated IL-1 secretion [22]. These data suggest that LPS and PMA are ideal substances to modulate the period and severity of the inflammation and resultant responses and the pattern of expressed and secreted mediators, respectively.

Among the analyzed cytokines and mediators are the proinflammatory marker IL-1 [23] and aggrecan (ACAN). ACAN, a major constituent of the ECM [24, 25], effectively neutralizes compressive forces, and that together with an elevated intra-articular ACAN level has been associated with cartilage damage [26].

Furthermore, the following mediators were included in our analysis; bFGF with its remodeling role in damaged tissue (especially in cartilage) [27, 28], the anabolic and cartilage protective factor IGF-1 and the regenerative factor BMP-7 [29, 30]. As these mediators were concomitantly determined in lavage fluids of acutely infected knee joints, our aim was to determine how reliably the* in vivo* situation can be reproduced* in vitro*. Can we determine early indicators* in vitro* and* in vivo* to improve diagnostic and therapy of joint infections, thus minimizing the need for surgery and helping to prevent cartilage damage at the same time?

## 2. Material and Methods

### 2.1. Isolation of Chondrocytes (CHDR)

Femoral heads were obtained during hip arthroplasty operations following femoral neck fractures. The degrees of osteoarthritis (OA) were evaluated on X-rays using Croft's modification of the Kellgren and Lawrence grading system. Cells from patients with advanced OA (≥grade 3) were not used for the experiments. Within 8 hours after surgery, the cartilage was separated from the bone, reduced to small pieces, and digested with collagenase CLS type II (Biochrom, Berlin, Germany) dissolved in Ham's F-12 medium with 10% FBS (Invitrogen, Karlsruhe, Germany) and 1% penicillin/streptomycin (Invitrogen, Karlsruhe, Germany). Following a 16-hour digestion period, cells were filtered, washed, and prepared for the experiments. The cells' preparation had been approved by the Ethics Committee of the University of Freiburg as part of the “Tissue bank for research in the field of tissue engineering” project (GTE-2002).

### 2.2. Isolation of Synovial Fibroblasts (SFB)

Synovia obtained during knee operations with an arthrotomy was kept buffered at 4°C. Within 8 hours after surgery, synovia was minced in pieces measuring about 3 × 3 mm followed by transfer into Ham's F-12 medium with 10% FBS (Invitrogen, Karlsruhe, Germany), 1% penicillin/streptomycin (P/S, Invitrogen, Karlsruhe, Germany), and 1% collagenase P (Roche, Mannheim). The suspension was digested in an incubator for 16 h at 37°C and 5% CO_2_. Liberated synovial fibroblasts (SFB) were filtered, washed, and seeded in expansion media (Ham's F-12, 10% FBS, 1 × P/S).

### 2.3. Isolation of Peripheral Blood Mononuclear Cells (MNC)

Human peripheral blood mononuclear cells were isolated from heparin-treated human whole blood by a Ficoll-Paque (Pharmacia, Piscataway, NJ, USA) gradient. The layer containing PBMCs was collected and washed three times with PBS. According to manufacturer's instructions, the cell population purified by this method consists of mainly lymphocytes and monocytes with little addition of granulocytes and erythrocytes.

### 2.4. 3D Coculture

#### 2.4.1. CHDR Embedding into Alginate Beads

One million chondrocytes (CHDR) were resuspended in 1 mL 1.2% alginic acid (Sigma, Taufkirchen, Germany) and transferred into a syringe with a 27 G needle (Braun, Melsungen, Germany). In order to polymerize stable CHDR/alginate beads, the cell suspension was dripped into 0.1 M CaCl_2_ solution. After 2 wash steps with PBS, the beads were cultivated for one day in Ham's F-12, 10% FBS, 1 × P/S, at 37°C, 5% CO_2_.

#### 2.4.2. SFB, CHDR, and MNC Culture

Two hundred thousand SFB per well in a 6-well plate were seeded and cultivated in Ham's F-12, 10% FBS, 1 × P/S, at 37°C, 5% CO_2_ for one day. The following day, around 20 alginate beads were given to the SFB per well. On the same day, freshly isolated MNC were adjusted to 1 × 10^6^ cells per mL and placed in cell culture inserts with 1 *μ*m pore size (BD, Heidelberg, Germany) and cultivated for one day in Ham's F-12, 10% FBS, 1 × P/S, at 37°C, 5% CO_2_ in 6-well plates. On the following day, cell culture inserts were placed into the CHDR and SFB coculture. Over the following 3 days, we carried out a preconditioning phase of the coculture. With the start of the experiment on day 0, the coculture was challenged with 250 ng/mL LPS (product number L6529, serotype* E. coli*. 055:B5, Sigma, Taufkirchen, Germany). On day 3, one half of the LPS challenged coculture was superchallenged by adding 100 ng/mL PMA (product number P1585, Sigma, Taufkirchen, Germany). On days 0, 3, and 7 half of the media was exchanged to minimize the inflammatory burden. This procedure was adapted to arthroscopic joint rinsing to minimize and halt the inflammatory burden. Media collected on days 0, 3, 7, and 10 were preserved at −80°C.

### 2.5. Clinical Evaluation

A consecutive series of 75 patients were recruited for the prospective collection of knee joint lavage fluid [31]. Inclusion criteria consisted of an arthroscopy of the knee joint because of an acute infection (confirmed macroscopically and microbiologically), informed consent to participate in the study, and aged between 18–70 years (MISSinG, DRKS 00003536). The 5 patients in the control group had no infection or cartilage lesions in MRI and diagnostic arthroscopy. Effusions were taken during arthroscopy and immediately kept frozen. Specimens were stored in liquid nitrogen until analysis. For model validation, 7 effusions were selected which fulfilled the conditions of a clearly defined starting point (all postoperative infections following arthroscopy) in basically healthy individuals (ASA 1 or 2). This setup ensured comparable conditions and a matching time frame for* in vitro* and* in vivo* analysis. The validation group's average age was 38 years ± 16 years, with a male/female ratio of 57/43%. The study was approved by the Ethics Board of the University of Freiburg (AN-EK-FRBRG-50/11). All patients participating in this study provided their written consent.

### 2.6. Analyses of Synovial Joint Fluids and Coculture Supernatants

#### 2.6.1. ELISA

Cytokines and proteins as IL-1, bFGF, IGF-1, ACAN, and BMP-7 detected in synovial joint fluids from acutely infected knee joints and coculture supernatants were analyzed by ELISA (RnD, Minneapolis, MN, USA) according to manufacturer's instructions. Briefly, this assay employs the quantitative sandwich enzyme immunoassay technique. The microplate was precoated with a specific monoclonal antibody. Supernatants were applied to the wells and, after washing, an HRP-conjugated specific antibody was added to the wells. Following the next wash, color development was proportional to the protein concentration and was calculated by comparison with a standard.

#### 2.6.2. Total Protein Determination

In order to normalize cytokine and mediators secreted in the synovial fluid as well as into the supernatant of the coculture, we divided cytokine and mediator concentrations by the amount of total protein present (TP) in synovial fluids and coculture supernatants using the Pierce BCA Protein Assay Kit (Thermo Scientific, Rockford, IL, USA). Briefly, standard and working reagents were prepared for microplate procedure as instructed by the manufacturer. Twenty-five microliters of standard and unknowns was pipetted into a 96-well microplate followed by adding 200 *μ*L to each well. The subsequent incubation at 37°C lasted 30 minutes (°C = degree Celsius). Measurements were taken at 562 nm in a TECAN Infinite M200 Reader.

### 2.7. Real Time PCR (RT-PCR)

RT-PCR was carried out for CHDR and SFB. RNA samples from days 0, 3, 7, and 10 were transcribed into cDNA; RNA analysis was carried out for gene expression of aggrecan, IL-1, BMP-7, bFGF, and IGF-1. Total mRNA was prepared using the Qiagen RNeasy kit according to manufacturer's instructions (Qiagen, Hilden, Germany). Total RNA (1* µ*g) was treated with 1 U DNAse I (Invitrogen, Karlsruhe, Germany) to remove genomic DNA. Poly-T primed cDNA synthesis was done using 1 U reverse transcriptase III (RTIII, Invitrogen) per 1 *μ*g RNA according to manufacturer's instruction. TaqMan PCR assays were performed in 384-well plates in a Roche LightCycler480 (Roche, Mannheim, Germany) using the Roche LightCycler Mastermix. For gene expression analyses, Roche's universal ProbeLibrary Probes and recommended Universal ProbeLibrary Reference Gene Assays were used. The 2-step cycling conditions were denaturation at 95°C for 5 min, followed by 45 amplification cycles at 95°C, 10 sec, 60°C, 35 sec, and 72°C 1 sec. Data was quantified via ΔΔCT comparisons. Data were normalized by comparing genes of interest versus reference genes (ACTB). Reaction efficiency is controlled by a relative standard curve and/or a calibrator per reaction.

### 2.8. Histological Examinations

#### 2.8.1. CHDR

CHDR/alginate beads from days 0, 3, 7, and 10 were preserved by fixation in PBS-buffered 2% formaldehyde for 24 h at room temperature, followed by fixation in 50 mM BaCl_2_/1% sucrose and 1% eosin for 30 minutes. Afterwards, CHDR/alginate beads were dehydrated by graded dehydration in isopropyl alcohol (75%, 96%, and 100%) for 10 min each, followed by paraffin embedding. To detect sulfated glycoproteins synthesized by CHDR, 5 *μ*m cuttings of CHDR/alginate beads were rehydrated in xylol and graded isopropyl alcohol (100%, 96%, and 75%) steps for 10 min each. After a short rinse in dH_2_O, incubation in 0.1 N HCl for 10 min followed by incubation in 1% Alcian/Blue/0.1 N HCl for 60 min took place. Counterstaining was carried out by nuclear fast red-aluminum sulfate solution 0.1% for 10 min followed by dehydration in graded isopropyl alcohol (75%, 96%, and 100%) and xylol for 5 min each and embedding in Entellan (Sigma, Taufkirchen, Germany). Image acquisition and analysis were done using Zeiss Axioplan 200 and Axiovision 4.6, respectively.

#### 2.8.2. SFB

SFB grown on cover slides in coculture approaches were fixed by PBS-buffered 2% formaldehyde for 24 h at room temperature, followed by giemsa staining for 10 min. Image acquisition and analysis were done by Zeiss Axioplan 200 and Axiovision 4.6, respectively. Cell numbers were evaluated by ImageJ (Wayne Rasband, NIH, http://imagej.nih.gov/ij/download). Colored pictures were converted to gray scale pictures. Background correction, scale adjustment, and watershed analysis (discrimination of adjacent cells) were carried out, followed by counting the numbers of particles larger than 50 *μ*m^2^. The correlation factor of 44.9 was obtained by dividing the sizes of growth area per well (9.62*e*−4 m^2^) in a 6-well plate by a factor through that of the section's image size (2.14*e*−5 m^2^).

### 2.9. Data Analysis and Statistics

ELISA and TP were analyzed according to the manufacturer's instructions (RnD, Minneapolis, MN, USA; Thermo Scientific, Rockford, IL, USA), creating a standard curve and reducing data using a four-parameter logistic (4-PL) curve fit by using GraphPad Prism 5 software (GraphPad Software, Inc., La Jolla, CA, USA). All values were expressed as mean ± standard deviation. Data sets were examined with one- and two-way analysis of variance, and individual group means were then compared with the unpaired Student's* t*-test. In case of unequal variances, we applied the Aspin-Welch test. Since* in vitro* data did not reveal a normal distribution, statistical significance was nonparametrically tested using the* U* test according to Mann and Whitney. Statistical significance was defined when *P* < 0.05.

## 3. Results

### 3.1. Acute Inflammatory Challenge Results in Comparable Inflammatory Responses* In Vivo* and* In Vitro*

To determine inflammatory mediators expressed upon acute bacterial infection in large human joints, we set up a coculture model containing crucial articular human components. The inflammatory burden of the coculture challenged with single inflammatory stimuli was consecutively relieved on days 3, 7, and 10. This procedure mimics the applied clinical treatment of a repeated lavage. Mediators present in the supernatant were analyzed on the gene expression level as well.

### 3.2. Challenge with LPS and LPS/PMA Results in a Timely Regulated Response of the Proinflammatory IL-1

**Figure 1:**
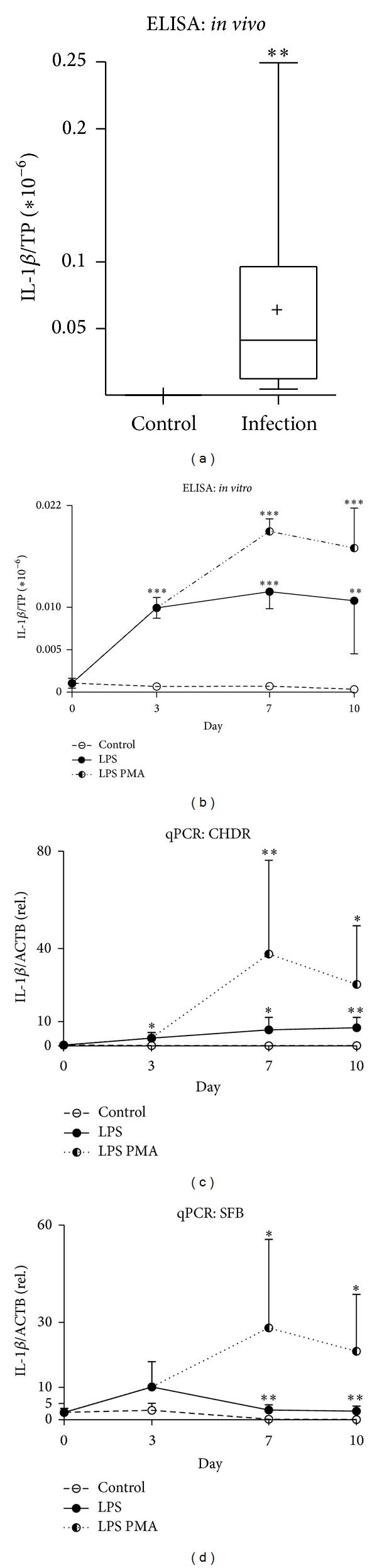
Detecting the inflammatory cytokine IL-1 in* in vivo* and in* in vitro* samples. Significantly elevated IL-1 level was detected by ELISA in lavage fluids from acutely infected knee joints ((a) **) as well as in cell culture supernatants upon an LPS challenge ((b) days 3, 7 ***, day 10 **P < 0.01) or LPS PMA ((b) days 7–10, ***). Accordingly, mRNA expression increased significantly in CHDR ((c) LPS: days 3, 7 *, day 10 **, LPS PMA: day 7 **, day 10 *) and in SFB ((d) LPS: days 7, 10 **, LPS PMA: days 7, 10 *). Statistics: (a) Mann-Whitney test and (b)–(d) Student's* t-*test, *P < 0.05, **P < 0.01, and ***P < 0.005. ELISA data were normalized versus total protein content (TP).

Inflammatory mediators formerly associated with altered cartilage biochemistry upon surgical [23] or chronic inflammation [32] were tested for their presence. In accordance with these findings, we observed significantly elevated inflammatory IL-1 (Figure 1(a), infection 0.07 ± 0.08; control 2.6 × 10^−^ ± 4.2 × 10^−7^) levels in synovial lavage fluids. Likewise, we noted significant increased IL-1 (*P* < 0.005) levels three days following the LPS challenge (Figure 1(b) 9.9 × 10^−9^ ± 0.5 × 10^−9^; control 0.6 × 10^−9^ ± 0.1 × 10^−9^). These concentrations remained at a significantly elevated level (PMA LPS, days 7–10, *P* < 0.005, 1.9 × 10^−8^ ± 0.1 × 10^−8^, 1.7 × 10^−8^ ± 0.5 × 10^−8^; controls 7.1 × 10^−10^ ± 1.3 × 10^−10^, 3.4 × 10^−10^ ± 0.9 × 10^−10^) which dropped slightly in LPS challenged samples (day 7, *P* < 0.005, 1.2 × 10^−8^ ± 0.2 × 10^−8^; day 10, *P* < 0.01, 1.0 × 10^−8^ ± 0.3 × 10^−8^). Regarding IL-1 mRNA expression, we detected a similar occurrence in the SFB and CHDR on day 3 (Figures 1(c) and 1(d)). We observed significantly elevated IL-*β* gene expression in CHDR stimulated with LPS from day 3 onwards (Figure 1(c), days 3–7, *P* < 0.05, 3.2  ±  2.2, 6.7 ± 5.1; day 10, *P* < 0.01, 7.5  ±  4.3; controls 0.20 ± 0.05, 0.08 ± 0.08, 0.1 ± 0.1). Gene expression following LPS PMA stimulation, however, peaked on day 7 and declined thereafter (Figure 1(c), days 3 and 10, *P* < 0.05, 25.3 ± 24.0; day 7, *P* < 0.01, 38.0 ± 38.0). Significantly elevated IL-1 mRNA expression in SFB was observed upon LPS (Figure 1(d), days 7–10, *P* < 0.01; 1.1 ± 0.6, 2.7  ±  1.6; controls 0.04  ±  0.02, 0.01  ±  0.01) and the LPS PMA challenge (Figure 1(d), days 7–10, *P* < 0.05; 12.1 ± 10.4, 9.6 ± 8.1).

### 3.3. The Homeostatic Cartilage Factor bFGF Senses Damage* In Vivo* as well as* In Vitro*

**Figure 2:**
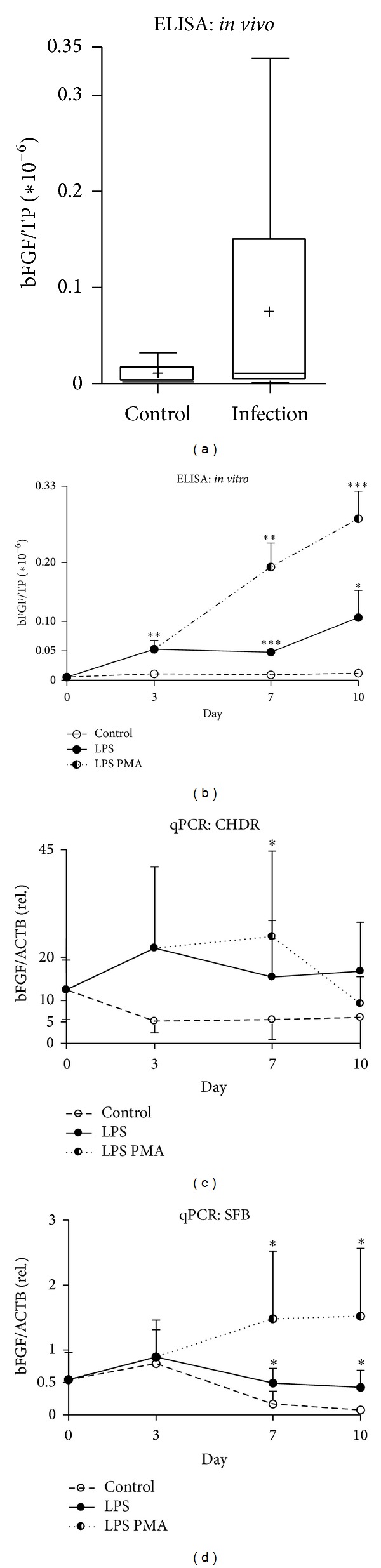
Detecting bFGF in* in vivo* and* in vitro* samples. Elevated bFGF levels observed in lavage fluids from acutely infected knee joints (a) which were significantly elevated in cocultures challenged by LPS ((b) day 3 *, day 7 ***, and day 10 *) or LPS PMA ((b) day 7 **, day 10 ***), too. Increased expression of bFGF mRNA was detected in CHDR and SFB upon LPS and LPS PMA challenge (c, d). Statistics: (a) Mann-Whitney test and (b)–(d) Student's* t*-test, *P < 0.05, **P < 0.01, and ***P < 0.005. ELISA data were normalized versus total protein content (TP).

We included in this analysis the growth factor bFGF, known as an intrinsic cartilage repair factor upregulated upon initial cartilage damage [27, 33]. We detected an increased bFGF level in lavage fluids (Figure 2(a), infection 8.3 × 10^−2^ ± 1.3 × 10^−1^; control 7.9 × 10^−3^ ± 1.4 × 10^−2^) and the coculture which persisted at a significantly elevated level from day 3, continuing through the coculture period (Figure 2(b), LPS, day 3, *P* < 0.01, 5.3 × 10^−8^ ± 1.5 × 10^−8^; day 7, *P* < 0.001, 4.8 × 10^−8^ ± 0.3 × 10^−8^; day 10, *P* < 0.05, 1.1 × 10^−7^ ± 0.5 × 10^−7^; LPS PMA, days 3–7, *P* < 0.01, 5.3 × 10^−8^ ± 1.5*E*-8, 1.9 × 10^−7^ ± 0.4*E*-7; day 10, *P* < 0.005, 2.7 × 10^−7^ ± 0.5 × 10^−7^; controls 1.1 × 10^−8^ ± 0.2 × 10^−8^, 9.3 × 10^−8^ ± 0.5 × 10^−8^, 1.2 × 10^−8^ ± 0.6 × 10^−8^). This is also reflected on gene expression in CHDR and SFB (Figures 2(c) and 2(d)). Correspondingly, from day 3 on, we noted a 3–5-fold rise in mRNA expression in LPS-stimulated CHDR. Similarly, a 3–5-fold increase in gene expression was observed in SFB treated with LPS on days 7 and 10 (Figure 2(d), day 10, *P* < 0.05, 0.4 ± 0.2; control 0.07 ± 0.05). Gene expression in CHDR challenged with LPS PMA exhibited a circa 5-fold upregulation on days 3 and 7 (Figure 2(c), LPS PMA, day 7, *P* < 0.05, 24.8 ± 19.9; control 5.5 ± 4.6). Even higher gene expression ratios were observed in SFB stimulated with LPS PMA on days 7 and 10 (Figure 2(d), *P* < 0.05, 1.3 ± 0.4, 1.3 ± 1.0; controls 0.16 ± 0.18, 0.07 ± 0.05).

### 3.4. Rise in the Anabolic and Protective Factor IGF-1 Correlates with Increasing Damage

**Figure 3:**
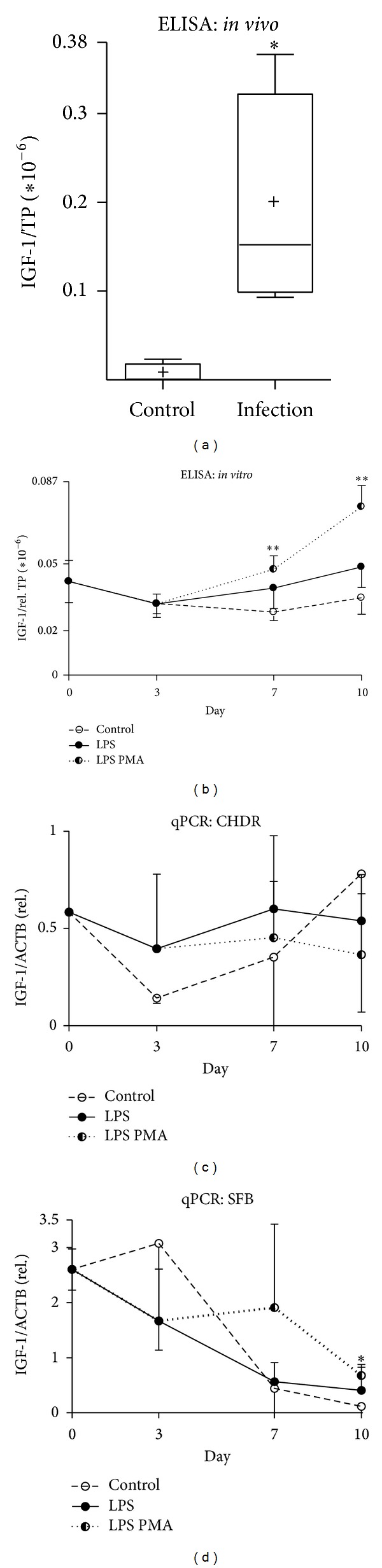
Detecting IGF-1 in* in vivo* and* in vitro* samples. Lavage fluids from acutely infected knee joints reveal elevated IGF-1 levels (a) also significantly elevated in cocultures challenged by LPS PMA ((b) days 7–10 **). No significantly altered IGF-1 mRNA expression in CHDR was apparent (c). After prolonged exposure to LPS PMA, however, significantly increased expression of IGF-1 mRNA was detected in SFB ((d) day 10 *); (a) Mann-Whitney test and (b)–(d) Student's* t*-test, *P < 0.05, **P < 0.01, and***P < 0.005. ELISA data were normalized versus total protein content (TP).

IGF-1 synthesized by many cells of mesenchymal origin is a major metabolic growth factor in articular cartilage [34, 35]. In addition, IGF-1 is able to lower the degradation of proteoglycan in cartilage exposed to IL-1 and TNF*α* [36]. We therefore included IGF-1 in this study to evaluate the effects of acute bacterial inflammation on IGF-1 expression. While we detected low IGF-1 levels in the lavage fluids of healthy patients, these levels were significantly elevated in infected joints (Figure 3(a), *P* < 0.05, infection 0.2 ± 0.1; control 7.5 × 10^−3^ ± 1.0 × 10^−2^).

In the coculture, we observed significant increased IGF-1 level after challenge with LPS PMA (Figure 3(b), days 7 and 10, *P* < 0.01; 4.8 × 10^−8^ ± 0.6 × 10^−8^, 7.6 × 10^−8^ ± 0.9 × 10^−8^; controls 2.8 × 10^−8^ ± 0.4 × 10^−8^, 3.5 × 10^−8^ ± 0.7 × 10^−8^). Despite different gene expression levels having been observed, CHDR expression did not result in a statistically significant upregulation upon inflammatory challenge (Figure 3(c)). However, we did observe significant different IGF-1 mRNA expression (around 6-fold) in the SFB after 10 days (Figure 3(d), *P* < 0.05, 0.7 ± 0.1; control 0.1 ± 0.1).

### 3.5. Elevated Liberation of Aggrecan following Acute Inflammation* In Vivo* and* In Vitro*

**Figure 4:**
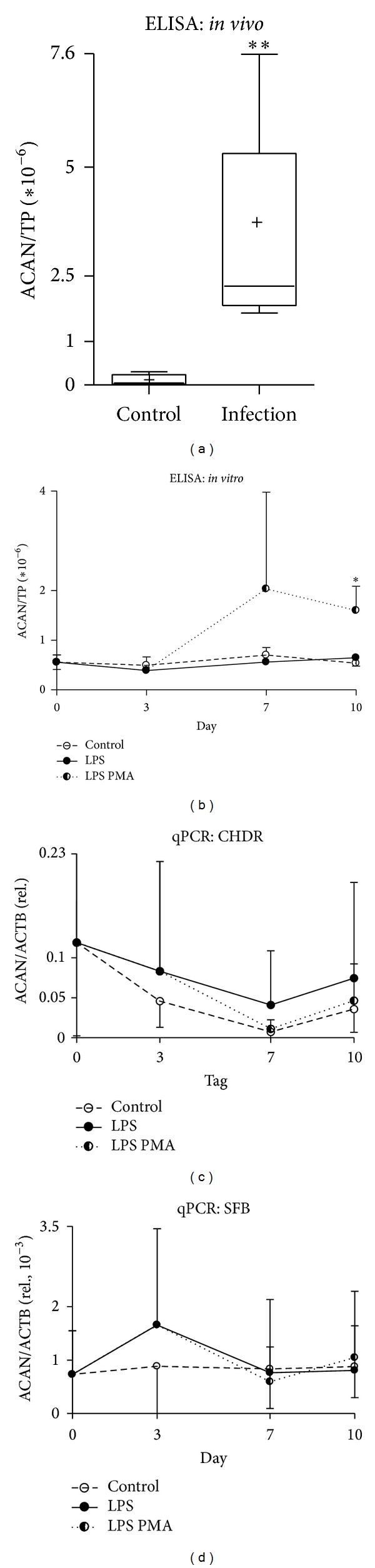
Detecting aggrecan in* in vivo* and* in vitro* samples. Significantly increased aggrecan (ACAN) levels were detected by ELISA in lavage fluids from acutely infected knee joints ((a) **). This was likewise observed in cocultures after prolonged LPS PMA challenge ((b) day 10 *). LPS and LPS PMA challenge results in slightly increased ACAN mRNA expression in CHDR ((c) days 3–10) and SFB ((d) day 3). Statistics: (a) Mann-Whitney test and (b)–(d) Student's* t*-test, *P < 0.05 and **P < 0.01. ELISA data were normalized versus total protein content (TP).

An elevated intra-articular ACAN level, formerly associated with cartilage damage [26], was detected in our analyses as well. Significantly elevated ACAN levels (Figure 4(a), around 30-fold, *P* < 0.01, infection 3.4 ± 2.2; control 0.1 ± 0.1) were observed in the lavage fluids from infected knee joints. We noted significantly elevated ACAN levels (3-fold) in the supernatants of the coculture after concerted application of LPS and PMA towards the end of the cultivation period (Figure 4(b), LPS PMA, *P* < 0.05, 1.6 × 10^−6^ ± 0.5 × 10^−6^; control 5.4 × 10^−7^ ± 0.5 × 10^−7^). The ACAN gene expression persisted throughout the observation period but the expression pattern did not correlate with increased ACAN protein levels (Figures 4(c) and 4(d)).

### 3.6. Cartilage Metabolic and Protective Factor BMP-7 Strongly Induced after Profound and Sustained Damage* In Vivo* and* In Vitro*

**Figure 5:**
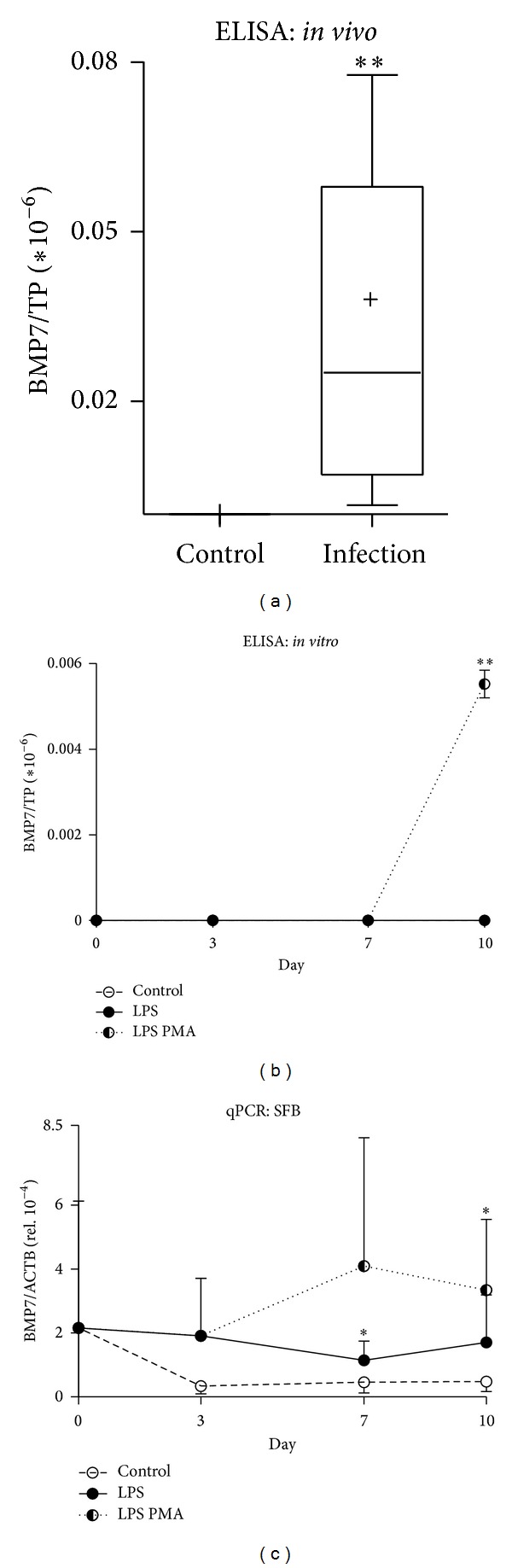
Detecting BMP-7 in* in vivo* and* in vitro* samples. Significantly elevated BMP-7 levels were detected by ELISA in lavage fluids from acutely infected knee joints ((a) **) also observed in cocultures after prolonged challenge with LPS PMA ((b) day 10 **). While no BMP-7 mRNA expression in CHDR was detected ((c) n.d.), a significant increase in BMP-7 mRNA expression was observed in SFB after either an LPS ((c) day 7 *) or LPS PMA challenge ((c) day 10 *). Statistics: (a) Mann-Whitney test and (b), (c) Student's* t*-test, *P < 0.05 and **P < 0.01. ELISA data were normalized versus total protein content (TP).

Osteogenic protein 1 (OP-1) or BMP-7, which is strongly associated with cartilage metabolism and boosts ECM synthesis, has the capacity to repair damaged cartilage [29, 37]. We noted significantly elevated BMP-7 levels in both lavage fluids (Figure 5(a), *P* < 0.01, infection 3.2 × 10^−8^ ± 3.0 × 10^−8^, control 1.8 × 10^−11^ ± 4.0 × 10^−11^) from infected joints and* in vitro*. Maximal inflammatory challenge with LPS and PMA resulted in significantly elevated BMP-7 levels on day 10 (Figure 5(b), LPS PMA, *P* < 0.01, 2.3 × 10^−11^ ± 0.2 × 10^−11^; control n.d.). In concert with an increased BMP-7 protein level, SFB challenged with LPS or LPS PMA manifested a rise in gene expression (Figure 5(c)) of 2.5-fold (LPS, day 7, *P* < 0.05, 1.1 × 10^−4^ ± 0.6 × 10^−4^; control 4.6 × 10^−5^ ± 3.4 × 10^−5^) and 7-fold (LPS PMA, day 10, *P* < 0.05, 3.8 × 10^−4^ ± 1.4 × 10^−4^; control 0.5 × 10^−4^ ± 0.3 × 10^−4^). We detected no BMP-7 gene expression in CHDR.

### 3.7. CHDR Remain Viable and Retain Ability to Synthesize Proteoglycans

**Figure 6:**
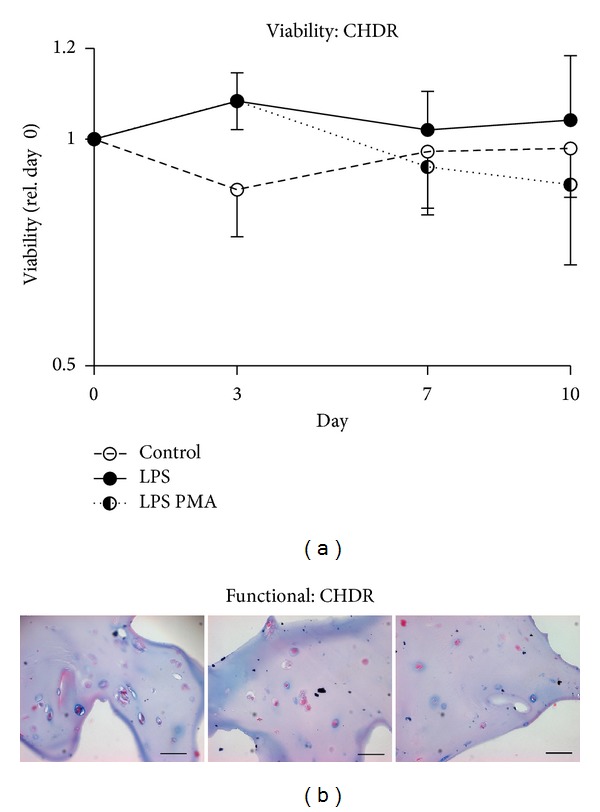
Viability and functionality of chondrocytes (CHDR). Cocultivated CHDR exposed to LPS, LPS PMA, or unchallenged were tested for their viability by MTS (a). While the LPS challenge tends to increase metabolic activity in CHDR, prolonged and severe inflammatory challenge by LPS PMA tend to result in diminished metabolic activity. However, we observed no significant differences in viability (a). Histological analyses by Alcian-Blue HCl staining for highly sulphated glycoproteins support these findings ((b) left: control; middle: LPS; right: LPS PMA challenge). Statistics: (a) Student's* t*-test. Scale bar 50 *μ*m.

Chondrocytes, the cells that synthesize and maintain cartilage, were analyzed for their ability to resist potentially damaging effects by maintaining their viability and functionality. For this, metabolic activity (by converting the MTS tetrazolium salt into formazan) was used to evaluate the viability of CHDR after an LPS and LPS PMA challenge (Figure 6(a)). Compared to controls, LPS-treated CHDR tend to increase their metabolic activity. LPS PMA-treated CHDR however exhibit diminished metabolic activity over time. Altogether, we observed no significant differences in viability and metabolic activity throughout the experimental procedure. With respect to functionality, CHDR were subject to Alcian-Blue staining for chondroitin sulphate (the major cartilage component in aggrecan) [13]. Compared to the controls (Figure 6(b), left), neither LPS (Figure 6(b), middle) nor LPS PMA (Figure 6(b), right) challenged CHDR lost their ability to produce sulfated proteoglycans at the end of the cultivation period.

### 3.8. Higher Inflammatory Challenge Causes a Decline in SFB Numbers

**Figure 7:**
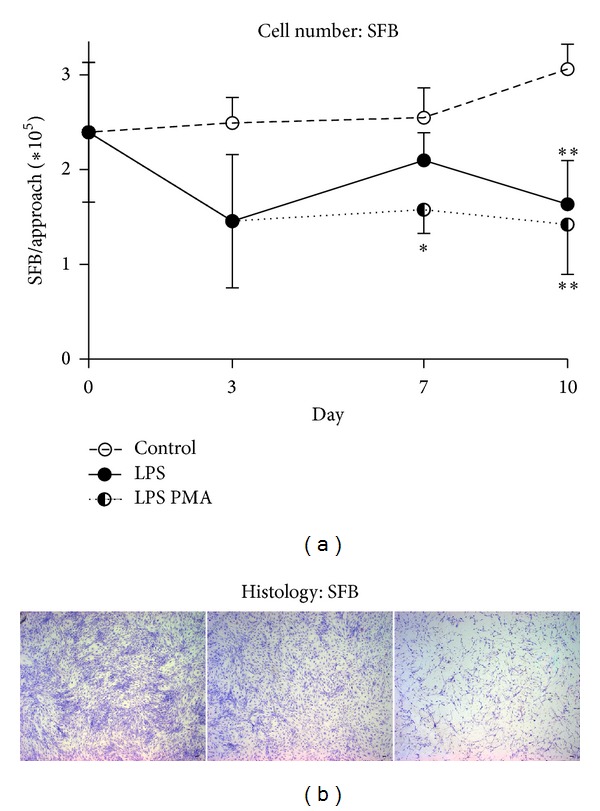
Synovial fibroblasts (SFB) proliferation. Originally in all cocultures, 2∗10^5^ SFB were seeded per well. While the SFB numbers increased steadily in controls, cell numbers in LPS decreased significantly (day 10, **P < 0.01) and LPS PMA assays (day 7, *P < 0.05; day 10, **P < 0.01). This observation is supported by giemsa-stained SFB on day 10 (b, left: control; middle: LPS; right: LPS PMA challenged cocultures). Statistics: (a) Student's* t*-test, *P < 0.05, **P < 0.01; cell number was determined by ImageJ. Scale bar 100 *μ*m.

While SFB numbers in control assays increased continuously, we noted a significant decrease in cell numbers in the LPS and LPS PMA challenged assays (Figure 7(a)). Whereas the number of SFB challenged with LPS exhibited a significant decline on day 10 (control 3.1 × 10^5^ ± 2.6 × 10^4^; LPS 1.6 × 10^5^ ± 4.6 × 10^4^; LPS PMA 1.4 × 10^5^ ± 5.2 × 10^4^), LPS PMA challenged SFB led to a significant decline in SFB numbers already from day 7 on (control 2.5 × 10^5^ ± 3.2 × 10^4^; LPS PMA 1.6 × 10^5^ ± 2.5 × 10^4^). This was confirmed by histological SFB findings on day 10.

## 4. Discussion

We developed and validated an* in vitro* model to reproduce the effects of acute inflammation in large human joints. The advantage of our model consists in monitoring impacts following acute inflammation over an extended time course including concurrent protein and its corresponding gene expression. The model compensates for the lack of concordance between protein and mRNA data. We observed that, after an initial release of proinflammatory IL-1 and catabolic bFGF, a delayed counterregulation of protective anabolic factors as BMP-7 takes place. We were thus able to demonstrate that both CHDR and SFB produce cartilage-specific mediators.

Longer-lasting inflammation inevitably results in a loss of cartilage integrity involving impaired joint function and ultimate destruction [38, 39]. Proinflammatory macrophage derived IL-1 [40] manifested significantly elevated levels in the synovial fluids of affected patients. The coculture reflected this as well. Here, we detected significantly elevated IL-1 levels following LPS and LPS PMA shots throughout the cultivation period. Superimposition of PMA to LPS-stimulated cocultures resulted in enhanced IL-1 secretion [22]. Whereas LPS primarily causes a sharp and transient release of IL-1, there is a longer-lasting response to PMA [21]. These two substances enabled us to modulate the severity of the inflammatory stimulus, especially regarding the deleterious effects on CHDR. In a clinical context, the extent of inflammation was determined by microbiologically analyzing lavage fluids.

Interestingly, the inflammatory stimulus was also apparent in an altered gene expression. We noticed significantly elevated IL-1 gene expression levels in CHDR and SFB. A further and long-lasting increase in both cell types was observed following PMA application. These results demonstrate that CHDR and SFB are not only affected by inflammation. Moreover, they actively support and maintain inflammation and thus can lead to remodulation [41] and finally destruction [42] of the cartilage. Bivalent effects of IL-1 on human CHDR matrix degradation have been reported elsewhere [41].

Elevated catabolic bFGF, associated with disturbed cartilage homeostasis [27, 43], was present in lavage fluids, which were significantly elevated* in vitro* following LPS and PMA challenge. This occurred successively and in a manner correlating with the amount of damage. It is conceivable that altered homeostasis culminates in remodelling processes in CHDR and SFB. Equally, following LPS and PMA stimuli, bFGF gene expression in CHDR increased up to fourfold, and about 20-fold in SFB.

Similar observations revealed that the ectopic expression of bFGF and EGF in CHDR resulted in increased proliferation and a concomitant decrease in collagen type II [33] and aggrecan [44] expression. A major function of CHDR, however, consists of ensuring cartilage homeostasis. But, differentiated CHDR possess a very limited capacity to proliferate. So, inducing proliferation [45] by enhancing bFGF synthesis might limit damaging effects.

Concomitant with increased bFGF levels, we noted significantly elevated IGF-1 levels in synovial lavage fluid as well as in LPS PMA challenged cocultures. Reports have shown that IGF-1 induces cartilage-specific induction of matrix synthesis [35] and prevents cytokine-stimulated degradation of proteoglycan in cartilage [36] to a certain extent. Supposedly, the progressive release of IGF-1 from the CHDR surrounding ECM into coculture supernatant is a means of antagonizing these effects. Thus, a stress-induced release of IGF-1 from the ECM precedes active counterregulation on gene expression level. An indication of this might be the fact that IGF-1 gene expression detected in CHDR and SFB did not correlate with protein secretion. Only over the long term did SFB, exposed to a stronger challenge, exhibit a significantly elevated IGF-1 mRNA level. An explanation for this may be either a regulatory mechanism or differences between IGF-1 gene expression and protein synthesis, as shown previously. On the other hand, SFB might contribute to minimizing deleterious effects when necessary. Nevertheless, a net loss of proteoglycan and the suppression of beneficial IGF-1 effects may occur. High bFGF levels were reported to shift the fine-tuning capabilities of cartilage to support ECM homeostasis versus a catabolic situation [28].

In this context we find the explanation for the significantly elevated levels of the proteoglycan ACAN detected in lavage fluids from acutely infected knee joints. Similarly, concerted application of LPS and PMA resulted in a three-fold increase in ACAN in supernatants of the cocultures as well. In line with our observations, the liberation of total or spliced ACAN following matrix breakdown during osteoarthritis progression has been described ACAN gene expression persisting throughout the experiment in CHDR and SFB did not correlate with an increased ACAN protein level. The disparate ACAN protein and gene expression may reflect a different regulatory mechanism. Or it might simply be evidence of uncontrolled liberation of ACAN owing to devastating effects during a massive inflammation as provoked by concerted LPS and PMA application.

The significantly elevated presence of the proanabolic [46] cartilage repair factor BMP-7 [29, 30] in the lavage fluids might indicate a repair attempt. In this regard, there is evidence that BMP-7 antagonizes the suppressive effects of LPS [47]. Furthermore, we observed significant BMP-7 levels in our cocultures after maximal inflammatory stimulus. Here, a significantly elevated BMP-7 level on day 10 might reflect the aforementioned situation. In addition, BMP-7 gene expression observed in SFB significantly rose in the course of inflammation caused by both LPS and LPS PMA. This in turn might also indicate that SFB or synovia assumes a repair function if, for example, CHDR are overwhelmed. During early events of an acute inflammation, a specific cytokine pattern in the joint cleft could initiate such a rescue operation to help cartilage to fend off damaging effects. For example, the exogenous addition of BMP-7 prevented cartilage loss and resulted in histological proof of equal amounts of this factor in cartilage and synovium [48, 49].

CHDR in cocultures retained their viability and functionality throughout the experiment. Whereas we observed a slight increase in metabolic activity after LPS stimulus, the addition of LPS PMA led to a gradual decline. In addition, Alcian-Blue staining confirmed that CHDR were capable of maintaining the synthesis of sulphated proteoglycans. Challenged SFB, however, decreased in cell numbers.

## 5. Conclusion

Our* in vitro* model enabled us to successfully monitor acute inflammatory effects on crucial components such as CHDR and SFB in large synovial joints over an extended period of time. By examining the synovial lavage fluids from acutely infected knee joints we were able to verify these results. In addition, by reproducing early inflammatory events on protein and gene expression levels, we can draw conclusions regarding early inflammatory situations* in vivo*. Moreover, we provide evidence that SFB express cartilage protective factor BMP-7 to counteract detrimental inflammatory effects.
